# The effect of thermoelectric craniocerebral cooling device on protecting brain functions in post-cardiac arrest syndrome

**DOI:** 10.3389/fcvm.2024.1502173

**Published:** 2025-01-09

**Authors:** Aydın Nadir, Deniz Kara, Ayda Turkoz

**Affiliations:** ^1^ Department of Cardiology, Faculty of Medicine, Bezmialem Vakif University, Istanbul, Türkiye; ^2^ Department of Anesthesiology and Reanimation, Faculty of Medicine, Bezmialem Vakif University, Istanbul, Türkiye

**Keywords:** cardiac arrest, hypothermia, neurological outcomes, post-resuscitation care, target temperature management

## Abstract

**Aim:**

This study aimed to protect brain functions in patients who experienced in-hospital cardiac arrest through the application of local cerebral hypothermia. By utilizing a specialized thermal hypothermia device, this approach sought to mitigate ischemic brain injury associated with post-cardiac arrest syndrome, enhance survival rates, and improve neurological outcomes as measured by standardized scales.

**Methods:**

A prospective, single-center cohort study was conducted involving patients aged ≥18 years who experienced in-hospital cardiac arrest and achieved return of spontaneous circulation (ROSC). Patients were cooled using a hypothermia helmet to achieve a target temperature of 32°C–34°C, maintained for 36–72 h, followed by controlled rewarming and normothermia for 72 h. Neurological recovery was assessed using the Cerebral Performance Category (CPC) scale, where CPC 1–2 denotes good recovery and CPC 3–5 indicates poor outcomes. Body temperature, hemodynamic parameters, biochemical changes, and survival data were meticulously recorded and analyzed. Statistical analysis included paired *t*-tests to compare pre- and post-treatment data.

**Results:**

Of 116 cardiac arrest cases, 30 (25.86%) were in-hospital, and 16 (53.33%) of these achieved ROSC. Among the patients, 62.5% underwent emergency coronary angiography due to ST-elevation myocardial infarction (STEMI). The mean time to hypothermia initiation was 32.9 ± 13.5 min, with hypothermia maintained for 58 ± 6.4 h. Neurological outcomes were favorable, with 62.5% of patients achieving CPC scores of 1 or 2, indicating functional recovery and independence. In contrast, CPC scores of 3 or higher were observed in 37.5% of patients, reflecting varying degrees of disability. Biochemical analysis revealed significant decreases in sodium, potassium, calcium, and magnesium levels, alongside increased urea and creatinine concentrations. Hemodynamic improvements included elevated systolic blood pressure and heart rate, while left ventricular ejection fraction remained stable. Overall survival was 75%, and the majority (62.5%) of survivors were discharged without significant neurological deficits.

**Conclusion:**

The findings suggest that early and targeted application of craniocerebral thermal hypothermia has the potential to improve survival and preserve neurological function in post-cardiac arrest syndrome. The high rates of favorable outcomes, as reflected by CPC scores, underscore the neuroprotective effects of localized hypothermia. Further large-scale, multicenter trials are recommended to validate these promising results and refine protocols for optimal clinical application.

## Introduction

According to the American Heart Association's 2019 updated statistics, approximately 565,000 cardiac arrest cases occur worldwide. Of these cases, 356,000 are out-of-hospital, while 209,000 are in-hospital. According to United States data, the survival rate is 12% for out-of-hospital cardiac arrest and 25% for in-hospital cardiac arrest. The survival rate with good neurological outcomes is 8% (1). Most of the poor outcomes and deaths of cardiac arrest survivors are attributed to widespread brain damage (2).

Therapeutic hypothermia was first applied in Russia in 1803 by covering the body with snow during the resuscitation of patients who developed cardiac arrest. In the 1958s, hypothermia was attempted by immersing the body in cold water during open heart, brain, and spinal cord surgeries. To increase the rate of sequela-free discharge of cardiac arrest survivors who can achieve rhythm with cardiac massage and to reduce brain damage, more modern, practical, fast, and easily applicable hypothermia devices are needed.

Organizations such as the International Liaison Committee on Resuscitation (ILCOR), American Heart Association (AHA), and European Resuscitation Council (ERC) state in their basic recommendations that “Adult patients showing ventricular fibrillation as the initial rhythm due to out-of-hospital sudden cardiac arrest should be cooled between 32°C and 34°C for 12–24 h” (3–5). Such a cooling method can also be beneficial in malignant arrhythmias such as VT/VF that primarily affect hemodynamics, post-CPR, and in-hospital sudden cardiac arrest patients. Today, hypothermia is accepted by medical science as a treatment method.

According to the guidelines published in 2022 by the European Resuscitation Council (ERC) and the European Society of Intensive Care Medicine (ESICM), adults who are unconscious after cardiac arrest should be treated with fever management and avoidance of fever. However, many questions about the optimal target temperature, cooling methods, and optimal duration still await answers (6).

In recent years, the use of therapeutic hypothermia in post-cardiac arrest patients has been a focus of clinical research. This study aims to contribute to the growing body of evidence regarding the potential benefits of craniocerebral hypothermia devices, which may offer a non-invasive and controlled method of cooling the brain following a cardiac arrest.

The aim of this study was to assess the safety, feasibility, and effectiveness of using craniocerebral thermal hypothermia as a treatment for patients with post-cardiac arrest syndrome. By utilizing a thermoelectric hypothermia helmet, the research sought to determine the device's ability to induce and maintain target temperature cooling (32°C–34°C) and its potential impact on improving neurological outcomes in this high-risk patient population.

## Methods

This study was a prospective, single-center cohort study conducted to assess the safety, feasibility, and potential impact of craniocerebral thermal hypothermia in patients with post-cardiac arrest syndrome. The cohort consisted of adult patients (≥18 years) who experienced in-hospital cardiac arrest and achieved return of spontaneous circulation (ROSC). The study spanned a period of 12 months, from January to December 2023.

### Study design

This study utilized a single-arm observational cohort design, aimed at evaluating the effects of a thermoelectric hypothermia device on targeted temperature management (TTM) and neurological recovery in patients following in-hospital cardiac arrest. Due to the absence of a control group, no comparative analysis was conducted. Instead, the The primary focus of this study was to evaluate the safety and feasibility of the intervention.

### Inclusion criteria

• Adults (≥18 years) with in-hospital cardiac arrest of cardiac origin (e.g., ventricular fibrillation, ventricular tachycardia, asystole, or unknown causes).
• Patients who achieved ROSC and spontaneous circulation.
• Glasgow Coma Scale (GCS) ≤9 upon admission.
• Patients who were intubated and eligible for therapeutic hypothermia.

### Exclusion criteria

• Age <18 years.
• Consciousness (GCS >9) at the time of admission.
• Active cancer treatment or severe comorbidities (e.g., acute stroke, advanced dementia, pregnancy).
• Body temperature <30°C at the time of arrival.

The primary objective of this study was to evaluate the effectiveness of craniocerebral hypothermia in improving neurological outcomes in post-cardiac arrest patients. Descriptive analysis of neurological recovery was performed using the Cerebral Performance Category (CPC) scale, assessed at multiple time points during and after treatment.

Due to the single-arm design, a comparative analysis against other interventions could not be performed. The study focused on describing changes in neurological status, including CPC scores correlated with different treatment phases: cooling, rewarming, and post-recovery.

Additionally, clinical parameters such as survival rates, time to normothermia, and biomarkers were tracked to further evaluate the potential impact of hypothermia on patient outcomes.

For patients who developed cardiac arrest, advanced life support protocols were promptly initiated, including intubation and cardiac resuscitation. Body temperature was continuously monitored using transnasal esophageal probes and axillary probes after intubation. Initial body temperature, hypothermia onset, duration, and time to normothermia were systematically recorded.

**Figure 1:**
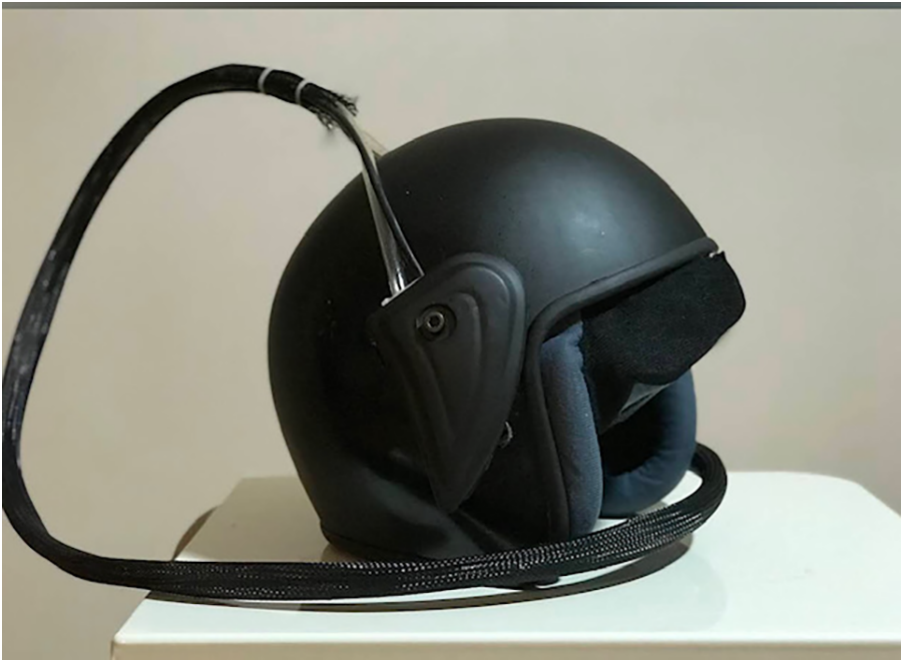
Thermoelectric hypothermia helmet.

**Figure 2:**
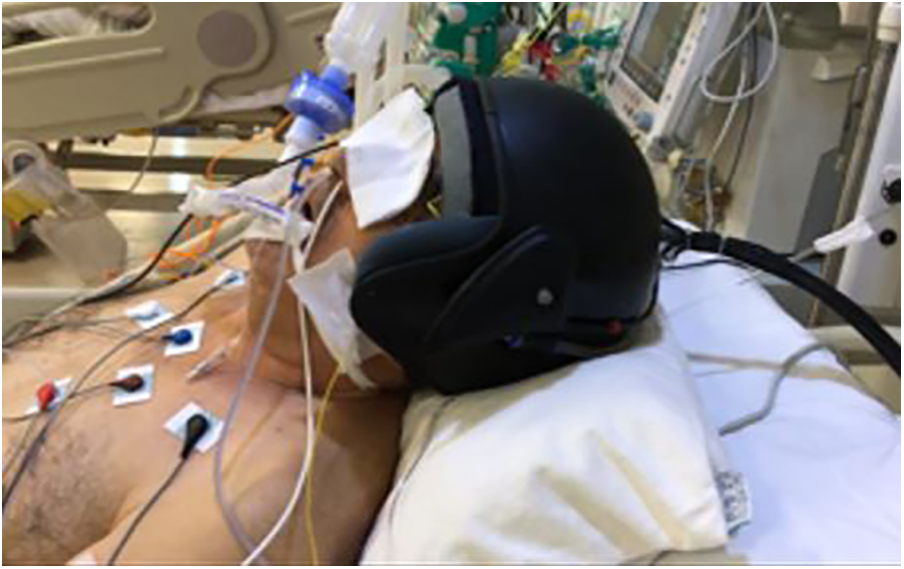
Example of thermohypothermia application in a patient.

The Turkish-patented thermoelectric hypothermia helmet used in this study was specifically designed for craniocerebral hypothermia (CSH) (7) (Figures 1, 2). Informed consent was obtained from all patients' families before initiating treatment.

Safety and Complications: The safety of the thermoelectric hypothermia device was closely monitored throughout the study, with particular focus on potential therapeutic hypothermia (TTM)-related complications.

Other potential complications associated with TTM, such as hypotension, electrolyte imbalances, and skin injuries (e.g., pressure sores from prolonged cooling), were closely monitored. Blood pressure, electrolyte levels, and skin integrity were regularly assessed, and corrective interventions were implemented when necessary. Arterial blood gases and fingertip blood glucose were monitored hourly to assess metabolic and respiratory status, ensuring the safe management of hypotension and other complications.

As a therapeutic hypothermia protocol, patients were gradually cooled at 0.5°C in 2 h to reach the target body temperature of 32°C–34°C in 2–4 h, and hypothermia was applied continuously for 36–72 h. Appropriate sedation was provided to patients during hypothermia. For sedation, Propofol and Remifentanil (Propofol 1 mg/kg bolus followed by 0.3–4.0 mg/kg/h, Remifentanil 0.05–0.1 mg/kg/min) were used according to the patient's weight. After therapeutic hypothermia, patients were started to be rewarmed at a rate of 0.25–0.50°C/h, and controlled normothermia was maintained for 72 h, followed by prognosis evaluation.

Glasgow Coma Scale and Cerebral Performance Category scale were used to evaluate patients' consciousness (8). Hourly arterial blood gas and fingertip blood sugar monitoring were performed during hypothermia. Patients' hemodynamic changes, recurrent arrhythmias, death, or discharge data were recorded. Hospital stay duration was recorded from the time of admission until discharge. The CPC score assessment was performed regularly during the hospital stay and at discharge to evaluate the neurological recovery process of the patients.

Fluid management strategies were employed to prevent hypotension during cooling and to ensure adequate organ perfusion. Adverse events, including complications arising from cooling and rewarming, were systematically recorded, and the safety profile of the device was assessed based on the incidence and management of these complications.

### Treatment timeline

1. Hypothermia Initiation (Time 0): Immediate initiation following cardiac arrest
2. Cooling Phase (36–72 h): Continuous hypothermic treatment
3. End of Cooling (72nd hour): Completion of cooling phase
4. Rewarming Phase (72 h): Gradual rewarming with maintained normothermia

### Smart thermoelectric hypothermia helmet

**Figure 3:**
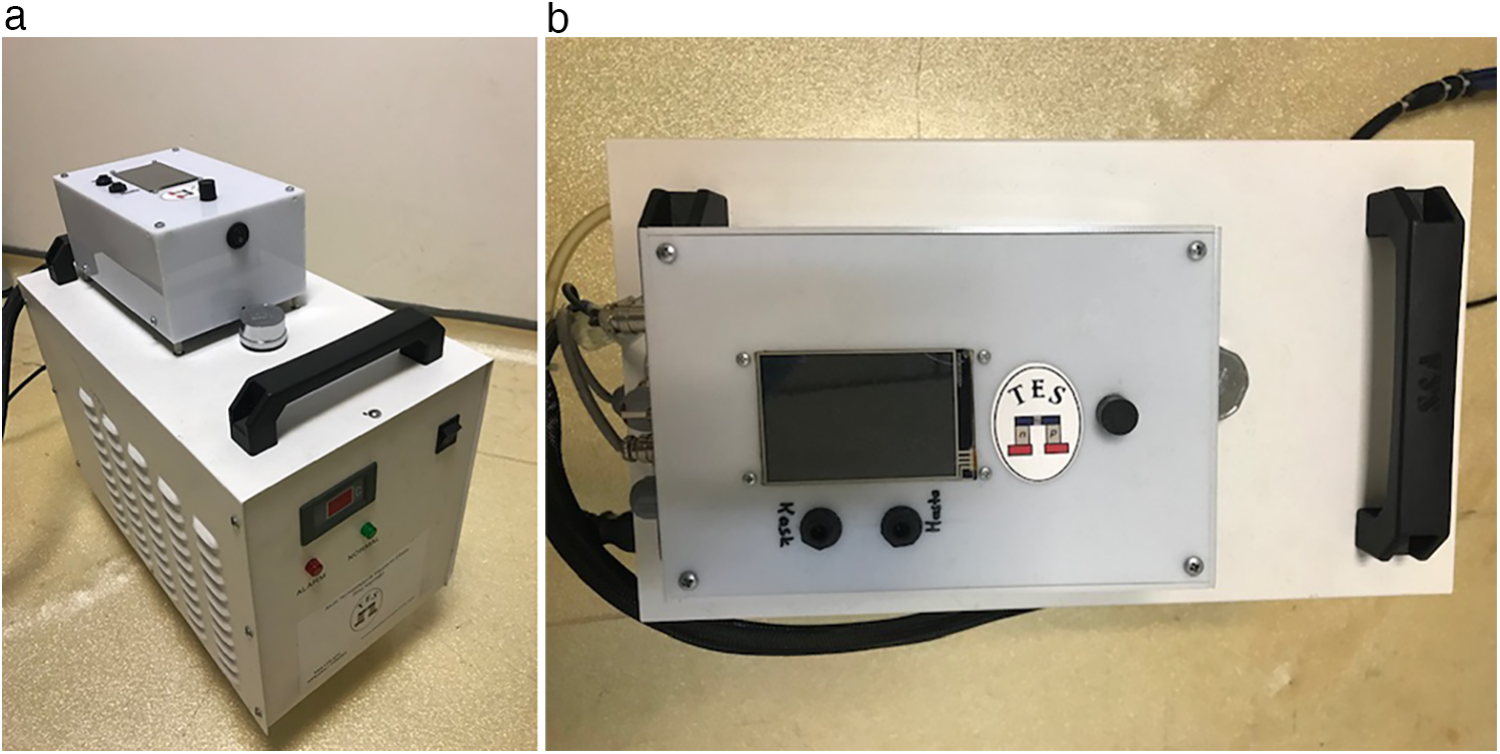
Thermohypothermia device. **(a)** Front view of the thermohypothermia device. **(b)** Top view of the thermohypothermia device.

The Thermohypotherm system, developed in Austria and patented by the Turkish Patent Institute (Figures 1, 3), is the first and only system of its kind, designed to induce both superficial and deep craniocerebral hypothermia. The thermoelectric micromodule, which is integrated into the helmet, facilitates external cooling of the head, thereby allowing the target brain temperature to be achieved by adjusting the applied DC current. By increasing the current, the cooling effect is intensified, and conversely, by reversing the direction of the current, the micromodule can act as a heater, enabling a controlled transition from hypothermia to normothermia once the desired cooling is achieved.

This system is integrated with both esophageal temperature and in-helmet temperature sensors, enabling continuous monitoring and automatic adjustment of the patient's body temperature. Through the device's digital interface, clinicians can set the target body temperature, define the maximum temperature drop allowed in the helmet, and control the duration of the hypothermic phase. In this study, the maximum temperature reduction achievable by the device was set at 20°C.

The thermoelectric hypothermia helmet employed in this study was designed to achieve controlled cooling specifically targeting the head region in patients with post-cardiac arrest syndrome. The in-helmet temperature represents the temperature measured within the space between the patient's skin and the helmet, influenced by the cooling effect of the integrated thermoelectric micromodule. While this measurement reflects the cooling efficiency of the helmet, it does not directly correlate with the brain temperature. Instead, it serves as an indirect indicator of the cooling effect on the brain. To standardize temperature management and align with conventional practices, esophageal temperature was utilized as the target body temperature in this study. This approach is consistent with widely recognized temperature management systems, such as Thermogard and Arctic Sun, which rely on continuous core body temperature monitoring as a standard for guiding therapeutic hypothermia. These systems ensure precise control over the cooling process, reducing variability and optimizing patient outcomes. Since direct measurement of brain temperature was not conducted, the expected brain temperature was inferred based on the combined analysis of in-helmet temperature and esophageal temperature, reflecting the potential impact of localized head cooling on cerebral thermoregulation.

### Cerebral performance category

It is a scale categorized between 1 and 5 points to evaluate patients' cerebral performance after CPR (8). Table 1 contains explanations about CPC. A CPC score of 1 or 2 was considered as survival with appropriate neurological function.

**Table 1:** Basic clinical and demographic characteristics of patients at the time of admission.

Parameter	Patients (*n* = 16)
Age	49.4 ± 7.3
Gender M, (*n*, %)	10 (62.5)
Obesity (*n*, %)	8 (50)
Hypertension (*n*, %)	8 (50)
Hyperlipidemia (*n*, %)	10 (62.5)
Diabetes (*n*, %)	4 (25)
Smoker (*n*, %)	10 (62.5)
Family history (*n*, %)	6 (37.5)
BMI (kg/m^2^)	23 5.2
ECG findings - NSR (*n*, %) - AF (*n*, %)	16 (100) 0 (−)

Table 1 Cerebral performance category (CPC).

CPC Description:

1. Return to normal cerebral functions and normal life
2. Independent daily life with limitations in brain functions
3. Inability to maintain daily life independently with severe effects on brain functions
4. Coma
5. Brain death

### Statistical analysis

Statistical analysis was performed using SPSS (Statistical Package for the Social Sciences) version 25.0. Continuous variables were expressed as means ± standard deviations (SD) and categorical variables as frequencies and percentages. Paired *t*-tests or Wilcoxon signed-rank tests were used to compare pre- and post-treatment biochemical markers and hemodynamic parameters. Survival analysis was performed using Kaplan-Meier curves to estimate overall survival and neurological recovery. A *p*-value < 0.05 was considered statistically significant for all analyses.

### Ethical considerations

The study was approved by the institutional ethics committee (approval number: 71306642-050.01.04/2020). Informed consent was obtained from all patients or their legal representatives prior to enrollment. This study adhered to ethical principles outlined in the Declaration of Helsinki.

## Results

During the 1-year study period, a total of 116 cardiac arrest cases were admitted to our clinic. Of the admitted patients, 86 (74.14%) were out-of-hospital arrests, and 30 (25.86%) were in-hospital arrests. Of the in-hospital arrests, 22 (73.33%) were cardiac, and 8 (26.67%) were respiratory in origin. Of the in-hospital cardiac arrests, 16 (72.73%) responded to CPR and sinus rhythm was achieved, while 6 (27.27%) patients did not respond to CPR and were lost.

Of the patients who achieved sinus rhythm, 10 (62.5%) had ST elevation on ECG [4 patients with inferior myocardial infarction (MI), 6 patients with anterior MI].These patients were taken for emergency coronary angiography. 6 (37.5%) patients had no ST elevation and did not undergo emergency coronary angiography.

A total of 16 cases, 10 (62.5%) of whom were male, were included in this study. All of the cardiac arrests occurred in the emergency department, and the cause of arrest was cardiac in all [2 patients with asystole (12.5%), 10 patients with ventricular tachycardia (62.5%), 4 patients with VF (25%)]. The basic clinical and demographic characteristics of the patients, characteristics and procedures related to cardiac arrest at admission are shown in Table 2.

**Table 2:** Characteristics and procedures related to cardiac arrest.

Parameter	*n*/Time
Cause of the cardiac arrest (*n*)	16
Cardiac (*n*)	16
Asystole (*n*)	2 (12.5)
Ventricular tachycardia (*n*)	10 (62.5)
Ventricular fibrillation (*n*)	4 (25)
Respiratory insufficiency (*n*)	–
Unknown	–
Cardiopulmonary resuscitation time (min)	16.3 ± 8.7
Hypothermia onset time (min)	32.9 ± 13.5
Time to reach target temperature 32–34°C (min)	214 ± 28.4
Duration of hypothermia <36°C (hours)	58 ± 6.4

10 (87.5%) patients in the angiography laboratory underwent interventional procedures. As a result of coronary angiography, critical lesions were detected in the right coronary artery (RCA) in 3 patients, in the circumflex coronary artery (Cx) in 1 patient, and in the left anterior descending artery (LAD) in 6 patients. TIMI III flow was achieved in all patients after the procedure. Table 3 shows the demographic characteristics of patients during the angiography laboratory.

**Table 3:** Coronary angiography and clinical parameters.

Parameter	Value (*n*, % or Mean ± SD)
Coronaryangiography	–
Electively (*n*, %)	10 (100)
Urgent (*n*, %)	−(0)
Diagnostic (*n*, %)	10 (100)
Interventional (*n*, %)	3 (30)
Inferior myocardial infarction	1 (10)
Right coronary artery (RCA)	6 (60)
Circumflex coronary artery (Cx)	
Anterior myocardial infarction	
Left anterior descending (LAD)	
Systolic blood pressure (mmHg)	143.8 ± 86.5
Diastolic blood pressure (mmHg)	78.3 ± 9.2
Heart rate (beats/min):	78.2 ± 11.2
Procedure time (min)	36.2 ± 8.6
Unfractionated heparin dose (U)	9,000 ± 1,000

**Figure 4:**
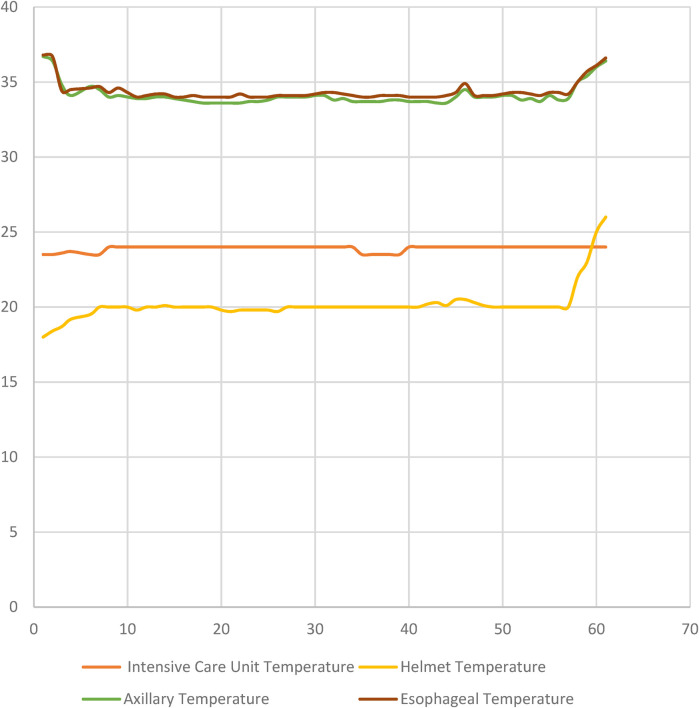
Temperature dynamics during hypothermic therapy in post-cardiac arrest patients are shown in Figure 4. The graph depicts temperature changes at various measurement sites (Intensive Care Unit, Helmet, Axillary, and Esophageal temperatures) over time, with measurements taken hourly and daily during the course of the therapy.

The mean CPR duration of the patients was recorded as 16.3 ± 8.7 min. The time to start hypothermia was determined as 32.9 ± 13.5 min, the time to reach the targeted body temperature was 214 ± 28.4 min, and the duration of hypothermia was 58 ± 6.4 h. Temperature changes during hypothermic therapy are shown in Figure 4. Figure 4 illustrates the temperature dynamics observed during hypothermic therapy in post-cardiac arrest patients, with measurements recorded hourly and daily at multiple sites, including the intensive care unit, helmet, axillary, and esophageal regions. This comprehensive monitoring allowed for a detailed evaluation of temperature changes throughout the course of therapy.

A significant temperature decrease was observed in both axillary and esophageal temperature follow-ups with the thermoelectric hypothermia device. The hypothermia helmet was found to be effective (*p* < 0.005). During hypothermia application, while a statistically significant decrease was observed in sodium, potassium, calcium, and magnesium levels in biochemical parameters, a statistically significant increase was observed in urea and creatinine values. Additionally, a statistically significant increase in systolic blood pressure and heart rate was determined with hypothermia. There was no statistically significant change in left ventricular Ejection fraction. (Table 4).

**Table 4:** Comparison of variables before and during hypothermia therapy.

Variable	Pre-HT (Mean ± SD)	During HT (Mean ± SD)	*p*-value
Sodium (mM/L)	134 ± 4.65	135 ± 3.98	0.103
Potassium (mM/L)	4.23 ± 0.65	3.96 ± 0.47	0.064
Calcium (mg/L)	7.83 ± 0.65	7.48 ± 0.58	0.075
Magnesium (mg/L)	2.14 ± 0.16	2,08 ± 0.13	0.087
Hemoglobin (g/dl)	12.8 ± 1.97	12,6 ± 1.69	0.308
Hematocrit (%)	39.1 ± 6.23	38,9 ± 5.04	0.174
Plt (103/ul)	310 ± 83	312 ± 87	0.751
Urea (g/L)	23.3 ± 8.7	25.7 ± 10.8	0.068
Creatinin (mg/dl)	1.06 ± 0.29	1.14 ± 0.38	0.074
Hs-CRP(mg/L)	14.83 ± 15.39	13.72 ± 13.4	0.589
Systolic BP(mmHg)	106.1 ± 13.01	114.4 ± 13.75	0.001
Diastolic BP(mmHg)	67.92 ± 10.19	70.88 ± 9.7	0.062
Mean arterial pressure (MAP)	80.64 ± 11.56	85.38 ± 11.72	0.031
Axillary temperature (C°)	36.2 ± 1.04	33.6 ± 1.06	0.001
Esophagus temperature (C°)	36.6 ± 1.12	34.1 ± 1.08	0.003
Temperature inside the helmet (C°)	20 ± 1.14	19 ± 1.17	0.084
Heart rate(beat/min)	116 ± 11	93 ± 10	0.034
EF%	58 ± 3.5	60 ± 2.1	0.291

“During HT” refers to the entire period of hypothermic therapy (HT), from the initiation of cooling to the end of the cooling phase. The mean and standard deviation (SD) values represent the data collected over this entire period.

Two patients lost their lives due to cardiogenic shock and re-arrest in the first hour of hospitalization. Hypothermia was terminated early in 6 patients due to GCS being above 9 in the first 24 h of hypothermia, and they were extubated by providing normothermia (GCS 15 and CPC 1). At the end of the 72nd hour, hypothermia was terminated for the remaining patients and status evaluation was made. Four patients demonstrated spontaneous breathing and a Glasgow Coma Scale (GCS) score above 9. Following clinical assessment and evaluation, extubation was performed on these selected patients after blood gas analysis and appropriate adjustment of ventilator settings. After extubation, the patients were evaluated with a GCS of 15 and a cerebral performance category (CPC) score of 4. Four patients could not be extubated as their GCS was below 9. In further follow-ups, 2 patients were lost due to pneumonia, and two patients were discharged with GCS 9 after tracheostomy was performed (Table 5).

**Table 5:** Clinical outcomes of hypothermia treatment in post-cardiac arrest patients.

Patient group	Initial clinical presentation (first 24 hours)	Clinical status after 72 hours of hypothermia	Extubation process	Final outcomes
Deceased patients	2 patients experienced cardiogenic shock followed by re-arrest and death			Mortality occurred within the first 24 h due to cardiogenic shock
Early termination of hypothermia	6 patients demonstrated GCS >9 within the first 24 h, hypothermia was terminated early, normothermia achieved (GCS 15, SPS 1)		Early extubation performed after reaching normothermia (GCS 15, CPC 4)	Successful recovery from hypothermia and extubation, no further complications noted
Gradual extubation group		4 patients exhibited spontaneous respiration and GCS >9 after 72 h of hypothermia	Gradual extubation performed post-hypothermia (GCS 15, CPC 4)	Successful extubation, with GCS 15, CPC 4; patients remained stable post-extubation
Non-extubation group		4 patients had GCS <9 after 72 h, failed to demonstrate spontaneous respiration	Extubation not performed due to poor neurological status	2 patients died from pneumonia; 2 patients discharged with tracheostomy and GCS 9

GCS, Glasgow coma scale score; CPC, cerebral performance category scale.

As a result, with early hypothermia application, the survival rate was 75%, while the rate of remaining without sequelae was 62.5%. The average hospital stay for surviving patients was 13 ± 7 days.

### Study population characteristics

The study included 16 patients, with 10 (62.5%) being male. All cardiac arrests occurred in the emergency department with the following rhythms:

• Asystole: 2 patients (12.5%)
• Ventricular tachycardia: 10 patients (62.5%)
• Ventricular fibrillation: 4 patients (25%)

### Coronary intervention outcomes

Among patients in the angiography laboratory:

• 10 patients (87.5%) underwent interventional procedures
• Critical lesions were identified in:
  ○ Right coronary artery (RCA): 3 patients
  ○ Circumflex coronary artery (Cx): 1 patient
  ○ Left anterior descending artery (LAD): 6 patients
• TIMI III flow was achieved in all patients post-procedure

### Effectiveness of hypothermia helmet

The effectiveness of the thermoelectric hypothermia helmet was primarily evaluated based on its ability to achieve and maintain the target temperature range of 32°C–34°C for the prescribed duration of therapy. A statistically significant decrease in body temperature was observed following the initiation of hypothermia treatment. The device effectively reduced body temperature, achieving the target range within an average of 214 ± 28.4 min, and maintained this temperature for 58 ± 6.4 h. The *p*-value of <0.005 was derived from a paired *t*-test comparison of pre-treatment baseline temperatures and post-treatment temperatures. This demonstrated that the helmet was statistically effective in reducing the core body temperature compared to baseline values.

Furthermore, the device demonstrated excellent control over temperature fluctuations during both the cooling and rewarming phases, with minimal variation from the target temperature. The controlled temperature regulation was essential in minimizing the risk of complications associated with uncontrolled hypothermia or hyperthermia.

### Lactate levels and cohort severity

Lactate levels, an important indicator of tissue hypoxia and severity of metabolic acidosis, were elevated upon admission for most patients, which is consistent with severe ischemia and shock at the time of cardiac arrest. Although lactate levels were not part of the routine study protocol, early lactate monitoring was conducted in a subset of patients, showing levels greater than 6 mmol/L in several cases. These elevated lactate levels gradually decreased with clinical stabilization and initiation of hypothermia treatment, reflecting improved tissue perfusion and metabolic recovery.

### Complications and device safety

No adverse events or device-related complications were reported throughout the study period, highlighting the safety of the thermoelectric hypothermia helmet in the clinical setting. Importantly, no skin injuries or pressure sores were observed, even after prolonged periods of hypothermia therapy. Continuous monitoring of the patients' body temperature ensured that the cooling process was precisely controlled, preventing the risk of overcooling or rebound hyperthermia. Furthermore, the helmet's low invasiveness was a key feature, as it did not require invasive procedures beyond routine clinical care, such as temperature monitoring via esophageal probes and arterial blood gases.

The safety profile of the device is a significant advantage, particularly in critically ill patients where the risk of complications from invasive procedures is high. The absence of complications associated with the device, combined with its ability to effectively induce and maintain hypothermia, suggests that the device could be a valuable tool in clinical practice for post-cardiac arrest care.

### Survival and neurological outcomes

• Survival rate: Overall, 75% of patients who achieved ROSC survived to discharge.
• Neurological outcomes: Among survivors, 62.5% had favorable neurological outcomes, with a Cerebral Performance Category (CPC) score of 1 or 2, indicating full or near-full recovery. These patients were able to return to independent daily living with minimal to no functional impairments. In contrast, 37.5% of survivors had a CPC score of 3 or higher, indicating moderate to severe neurological impairment. These patients were unable to perform activities of daily living independently and had ongoing cognitive or motor deficits.

## Discussion

In this study, we sought to investigate the neuroprotective effects of craniocerebral thermal hypothermia in patients who developed post-cardiac arrest syndrome. Our results indicated that early application of hypothermia led to a survival rate of 75%, with 62.5% of patients remaining without significant sequelae. These findings suggest that craniocerebral hypothermia may represent an effective strategy in mitigating brain damage following cardiac arrest.

The neuroprotective effects of mild hypothermia, particularly in alleviating global cerebral hypoxia and ischemic injury, have been well-documented in the literature (9, 10). Studies have consistently shown that therapeutic hypothermia improves neurological outcomes in patients post-cardiac arrest. The favorable survival and low sequelae rates observed in our cohort further substantiate these findings. Hypothermia exerts a protective effect by reducing cerebral oxygen consumption, which is critical in mitigating the deleterious consequences of hypoxia. A decrease of 1°C in body temperature results in approximately a 6% reduction in cerebral metabolic rate (11), thereby attenuating the formation of excitotoxic amino acids and free radicals. Moreover, hypothermia inhibits the release of intracellular excitotoxins and diminishes the inflammatory cascade that contributes to post-cardiac arrest syndrome (7, 12).

Although the systemic application of hypothermia has been associated with a range of complications, including arrhythmias, coagulopathies, and infections (13), our approach of local cerebral hypothermia seeks to minimize these risks. The craniocerebral thermal hypothermia device utilized in our study is an original, patented technology in Turkey (14), offering a more efficient and controlled cooling method compared to conventional systemic cooling techniques. In support of our clinical findings, animal studies have demonstrated favorable outcomes with similar hypothermia interventions. For instance, a study conducted on rodents revealed a dramatic 80% reduction in mortality following hypothermic treatment (15). While these results align with the positive outcomes observed in our clinical cohort, caution is warranted in extrapolating animal model data to human physiology due to inherent differences between species. Timing is a critical factor in the effectiveness of hypothermia in post-cardiac arrest patients. The literature emphasizes that early initiation of hypothermic therapy significantly improves neurological outcomes (16, 17).

Our study is a single-arm, non-randomized investigation, which is a key limitation when interpreting the results. The absence of a control group means that we cannot definitively attribute the observed outcomes solely to the device. However, the findings, particularly the neurological outcomes measured by the Cerebral Performance Category (CPC) scale, provide valuable insight into the potential benefits of localized hypothermia. Among survivors, 62.5% achieved CPC scores of 1 or 2, indicating favorable neurological recovery with minimal cognitive or motor impairment. These results suggest that localized cooling may contribute to preserving brain function following cardiac arrest, as supported by similar findings in other studies investigating hypothermic therapies.

The craniocerebral thermal hypothermia device also demonstrated an excellent safety profile. No adverse events related to the device, such as skin injuries, pressure sores, or hypothermic complications, were reported, supporting the non-invasive nature of the device. This is consistent with findings from other studies that have highlighted the low risk of complications associated with localized cooling techniques compared to more invasive cooling methods.

In our study, hypothermia was initiated on average within 32.9 ± 13.5 min after cardiac arrest, a factor we believe contributed significantly to the favorable results observed. The average duration of hypothermia in our cohort was 58 ± 6.4 h, which aligns with the 24–72 h duration recommended in prior studies (18, 19). However, further investigation is required to determine the optimal duration of hypothermia for maximal neuroprotection. Recent randomized controlled trials, such as the TTM2 trial, have raised questions regarding the precise temperature target for therapeutic hypothermia post-cardiac arrest (20). For example, the TTM2 study could not show a clear difference in whether target temperature management is more effective at 33°C or 37°C (21). However, local cerebral hypothermia applications like our study can add a new dimension to these discussions. Local application can provide deeper and more sustainable hypothermia in brain tissue while reducing systemic side effects. The findings from this study suggest that craniocerebral thermal hypothermia is a feasible and safe method for achieving controlled hypothermia in patients with post-cardiac arrest syndrome. However, it is important to note that due to the single-arm design and absence of a control group, this study was not designed to assess the definitive efficacy of the device on survival rates or neurological recovery. Therefore, conclusions regarding the device's effectiveness in improving outcomes should be made cautiously.

The primary objective of this study was to evaluate the safety, feasibility, and efficacy in achieving targeted hypothermia using the thermoelectric hypothermia helmet. The device successfully achieved and maintained the target temperature range of 32°–34°C, which is critical for neuroprotection in post-cardiac arrest syndrome. The precise and controlled temperature regulation within the helmet and systemically is a significant advantage, as it minimizes the risk of complications associated with more invasive cooling methods. In this cohort, patients were cooled effectively, with an average time of 214 ± 28.4 min to reach the target temperature, and cooling was maintained for 58 ± 6.4 h.

The favorable neurological outcomes, as measured by the Cerebral Performance Category (CPC) scale, showed that 62.5% of survivors achieved CPC scores of 1 or 2, indicating favorable neurological recovery with minimal cognitive or motor impairment. However, 37.5% of patients exhibited moderate to severe neurological impairments (CPC scores of 3 or higher), which is consistent with the known challenges of post-cardiac arrest syndrome.

It is important to emphasize that the absence of a control group limits our ability to directly attribute these positive neurological outcomes to the device alone. The observed results may also be influenced by the clinical interventions provided, the timing of resuscitation, and other patient-specific factors such as comorbidities and the severity of initial cardiac arrest.

The safety profile of the device is a key finding in this study. No adverse events related to the device, such as skin injuries, pressure sores, or hypothermic complications, were observed. This highlights the low invasiveness of the device, as it does not require invasive monitoring beyond standard clinical care procedures. The absence of any significant complications suggests that craniocerebral thermal hypothermia could be a promising non-invasive treatment method for post-cardiac arrest care, particularly in settings where other forms of therapeutic hypothermia might pose higher risks or be less feasible.

Despite these promising findings, it is essential to acknowledge the study's limitations. The small sample size, single-arm design, and lack of long-term follow-up restrict the ability to generalize the results. Therefore, the conclusions drawn from this study should be considered preliminary. Further large-scale, multi-center randomized controlled trials are needed to rigorously assess the device's impact on neurological recovery, long-term survival, and quality of life. Additionally, studies evaluating optimal cooling duration, cooling rate, and patient subgroups that may benefit most from this approach are warranted.

In conclusion, this study demonstrates that craniocerebral thermal hypothermia, using a thermoelectric hypothermia helmet, is a safe and feasible intervention for inducing controlled hypothermia in post-cardiac arrest syndrome. While the results suggest the device's potential for achieving the target temperature safely, the impact on neurological outcomes, as indicated by CPC scores, remains an important area for future investigation.

### Limits of the study

First, our number of patients is relatively small and there is no control group. This limits the generalizability of our results. Also, long-term follow-up results have not yet been obtained. Longer follow-up is required for a full assessment of neurological recovery.

In the future, the craniocerebral thermal hypothermia device should be further developed and its widespread use in ambulances and intensive care units should be targeted. This technology can be evaluated as a potential treatment method not only after cardiac arrest but also for traumatic brain injury, stroke, and other neurological emergencies.

## Conclusion

The results of this study suggest that craniocerebral thermal hypothermia is a potentially effective and safe intervention for achieving targeted therapeutic hypothermia in patients with post-cardiac arrest syndrome. While the study demonstrated favorable survival rates and promising neurological outcomes, the single-arm desig Primary focus was on n, lack of a control group, and relatively small sample size limit the ability to draw definitive conclusions regarding the device's impact on clinical outcomes. Therefore, caution is warranted when interpreting these findings as conclusive evidence of the device's efficacy in improving neurological recovery or long-term survival.

The primary strength of this study lies in its demonstration of the feasibility and safety of utilizing the thermoelectric hypothermia device in a clinical setting. The device successfully achieved the target temperature range (32°–34°C) in a controlled and sustained manner, which is essential for mitigating ischemic injury and reducing the effects of post-cardiac arrest syndrome. However, the observed improvements in survival and neurological recovery should be considered preliminary and indicative of the device's potential, rather than definitive evidence of its clinical efficacy.

Given the inherent limitations of the study, including the absence of a control group and the lack of long-term follow-up data, future research should prioritize the design of large-scale, multi-center randomized controlled trials (RCTs). These trials would provide a more comprehensive understanding of the device's clinical efficacy, specifically assessing long-term neurological recovery, survival rates, and any potential adverse effects. Further investigations are also needed to determine the optimal duration of hypothermia therapy and identify specific patient subgroups that may benefit the most from this approach.

In this study, patients who survived cardiac arrest and achieved favorable neurological outcomes were initially described as having recovered without sequelae. However, this term can be ambiguous and subject to interpretation. To provide greater clarity, we used the Cerebral Performance Category (CPC) scale to more precisely assess neurological outcomes. A CPC score of 1 or 2 indicates favorable recovery, with CPC 1 representing full recovery or mild impairments, and CPC 2 indicating moderate disability that does not hinder independent living. Therefore, rather than using the imprecise expression “without sequelae,” we now refer to CPC scores for a more specific and reliable evaluation of neurological function.

In conclusion, while this study contributes to the growing body of evidence supporting the use of localized craniocerebral hypothermia, further rigorous research is necessary to fully assess its role as a standard treatment for post-cardiac arrest syndrome.

## Data Availability

The original contributions presented in the study are included in the article/Supplementary Material, further inquiries can be directed to the corresponding author.
